# Association of lipoprotein(a), oxidized phospholipids and apolipoprotein B100 in acute ischemic stroke cohort

**DOI:** 10.70401/alr.2026.0005

**Published:** 2026-01-21

**Authors:** Yihao Li, Emmie Then, Salwa Rahman, Nelsa Matienzo, Anastasiya Matveyenko, Marianna Pavlyha, Farid Khasiyev, Randolph S Marshall, Joshua Willey, Jose Gutierrez, Gissette Reyes-Soffer

**Affiliations:** 1 Department of Medicine, Division of Preventive Medicine and Nutrition, Columbia University Vagelos College of Physicians and Surgeons, 630 West 168th Street, P & S 8-503, New York, NY 10032, USA.; 2 Department of Neurology, Division of Stroke and Vascular Diseases, Columbia University Vagelos College of Physicians and Surgeons, New York, NY 10032, USA.; 3 Department of Neurology, University of Miami Miller School of Medicine, Miami, FL 33136, USA.

**Keywords:** Lp(a), acute ischemic stroke, oxidized phospholipids

## Abstract

**Aims:**

Atherosclerosis, affecting the aorta, cervical, or intracranial arteries, is a common cause of stroke. Previous studies have shown a strong link between high Lp(a) levels and atherosclerotic stroke due to intracranial atherosclerotic disease, implicating Lp(a) in disease development and progression. The precise role of Lp(a) in stroke subtypes remains unclear, although smaller isoform sizes and oxidized phospholipids on Lp(a) are associated with the disease presence. To clarify Lp(a)’s connection with ischemic stroke subtypes, we evaluated various plasma biomarkers previously linked to Lp(a) and disease.

**Methods:**

We used stored plasma samples and data from 244 participants enrolled in an acute ischemic stroke registry at Columbia University Medical Center in New York. Plasma Lp(a) concentrations, apolipoprotein B100 (APOB), and oxidized phospholipids were measured via enzyme-linked immunosorbent assay. APO(a) isoform size was measured via gel electrophoresis. Stroke subtypes were classified based on etiologies using clinical and imaging data. Adjusted multivariate logistic regression models were built to assess associations between Lp(a)-related biomarkers and stroke subtype.

**Results:**

In participants with acute ischemic stroke, high Lp(a) concentrations, percentage of APOB in Lp(a), and OxPL-APO(a) concentrations were significantly associated with the presence of atherosclerotic stroke compared to those with non-atherosclerotic strokes [OR = 1.30 (*p* = 5.7e - 3), 1.29 (*p* = 6.9e - 3), 1.27 (*p* = 1.7e - 2), respectively]. In participants with atherosclerotic stroke, these changes were significantly associated with extracranial atherosclerotic stroke (ECAD), with an OR = 0.69, *p* = 4e - 2.

**Conclusion:**

In addition to Lp(a) concentrations, the percentage of APOB in Lp(a), and OxPL-APO(a) concentrations are positively associated with acute atherosclerotic ischemic stroke, specifically ECAD.

## 1. Introduction

Stroke is one of the leading causes of death and long-term disability^[1]^. Although there were some confounding studies showing no associations between stroke and high Lipoprotein(a) [Lp(a)] levels^[2,3]^, recent literature strongly supports the role of high Lp(a) in ischemic stroke^[4-9]^. Lp(a) concentrations tend to be higher in participants who have experienced a stroke compared to healthy, non-stroke individuals^[10,11]^. Importantly, most strokes are caused by ischemic events^[12]^. In the 2024 guideline for the primary prevention of stroke, the AHA/ASA recommends assessing certain genetic factors that increase stroke risk, which includes evaluating Lp(a) levels^[12]^. A recent meta-analysis^[13]^ shows that high Lp(a) levels are significantly associated with increased stroke recurrence (OR = 1.69; 95% confidence interval [CI]: 1.09-2.63; *p* = 0.020) and poor functional outcome (OR = 2.09; 95% CI: 1.40-3.11; *p* < 0.001).

Larger studies, including data from the UK biobank, have shown positive associations between high Lp(a) and ischemic stroke but not hemorrhagic stroke, mostly in men^[14]^. Other authors have found that high Lp(a) concentrations are associated with an increased risk of intracranial atherosclerotic disease (ICAD), but a decreased risk of small vessel stroke^[15]^. Moreover, a meta-analysis and further studies strongly support Lp(a) as an independent risk factor for ischemic stroke, specifically large artery disease/ICAD^[1,16,17]^. The mechanisms driving Lp(a)’s link to the development of acute ischemic stroke (AIS) are yet to be determined. Although current guidelines recommend use of Lp(a) measurements in those at high risk for atherosclerotic cardiovascular disease (ASCVD)^[18]^, including those with stroke, studies have highlighted that there is poor implementation of these practices^[19,20]^.

Lp(a) is an apolipoprotein B100 (APOB) containing lipoprotein. Unlike other APOB-containing lipoproteins, analysis of its protein components links it to pathways of atherosclerosis but also coagulation and inflammation^[21]^. Apolipoprotein(a) [APO(a)], the other major protein component of Lp(a), contains a multi-kringle (K) glycoprotein with varying molecular weight (200-800 kD). This large size polymorphism is determined by the Kringle IV type 2(KIV-2) repeats number in the *LPA* gene. Typically, individuals express two APO(a) isoforms. Small size APO(a) isoforms are associated with high plasma concentrations and vice versa^[21]^. Circulating Lp(a) particle concentrations are highly heterogeneous, widely right-skewed, and contain interindividual and interethnic variation, which is most likely due to *LPA* gene variability and APO(a) isoform size^[22-24]^. Previous literature supports a strong positive association between small apo(a) isoforms and cardiovascular disease^[25-27]^, coronary heart disease and ischemic stroke^[7,28,29]^. Additionally, studies have shown that Lp(a)’s correlation with increased atherogenicity and inflammation is mediated via oxidized phospholipid (OxPL)^[30,31]^. OxPL can be measured on APOB lipoproteins (OxPL-APOB) and on APO(a) [OxPL-APO(a)] using the E06 murine monoclonal antibody, which recognizes the phosphocholine head group of OxPL. Studies have implied that elevated OxPL-APOB levels are predictors of the diagnosis or prognosis of CVD, and it has been postulated that Lp(a) particles are the main carrier of OxPL when compared to LDL-APOB^[31-34]^. Although there exist parallels between coronary atherosclerosis and atherosclerosis elsewhere, the role of Lp(a) and related profiles is less clear in stroke populations. Therefore, we wanted to explore Lp(a) levels plus additional biomarkers and their relationships to subtypes of AIS groups. Thus, we provide novel data on the associations of Lp(a), APO(a) isoform size, APOB, and oxidized phospholipids in AIS; these measurements are not part of routine clinical care.

## 2. Methods

### 2.1 Study design and population

We performed a retrospective cross-sectional study. We used existing data and stored human blood samples to measure biomarker levels from the ongoing New York Presbyterian Hospital/Columbia University Irving Medical Center Stroke Registry Study (NYP/CUIMC-SRS)^[35]^. Research participants were enrolled when admitted to NYP/CUIMC with a clinical suspicion of stroke or transient ischemic attack. The primary goals of the registry are to assess the prevalence of vascular risk factors, the incidence of vascular outcomes, and to identify prevention strategies for stroke.

All acute ischemic stroke cases had their diagnosis confirmed by computed tomography and/or magnetic resonance imaging and were assessed by a qualified vascular neurologist. Additionally, only participants whose plasma samples were available, safely stored (stored in −80 °C freezer, never thawed, and stored for less than 5 years), and who had provided consent for future research were included. All baseline characteristics were collected upon study enrollment, including plasma lipids. The complete protocol was approved by the Columbia University Medical Center Institutional Review Board (IRB AAAS5563). Following these criteria only 3 individuals were excluded.

### 2.2 Baseline characteristics

Participants’ research registry records were reviewed to extract demographic variables: vital signs, vascular risk factors, comorbidities, pre-treatment NIH stroke scale, stroke etiology, age, sex, and self-reported ethnicity. The population’s ethnicity was categorized into Hispanics, Non-Hispanic Whites, Non-Hispanic Blacks, and others. Self-reported medical history included hypertension, diabetes, and hypercholesterolemia. Medication usage and additional measured biomarkers were collected during enrollment. Stored plasma and serum samples were isolated via centrifugation from blood samples collected within the first 7 days of hospital admission, in EDTA-containing tubes.

Stroke etiologies were adjudicated based on admission and inpatient workup. Clinical, laboratory, and imaging modalities were used to ascertain the most probable cause of stroke. All acute stroke cases did not have conflicting etiologies and had stored frozen plasma aliquots. The trial of Org 10172 in Acute Stroke Treatment classification system^[36]^ was utilized to ascertain etiology but was further refined by anatomical locations of presumed causes of large artery strokes (aortic, cervical or intracranial). Stroke etiology was extracted from the discharge summary of participants with stroke and further confirmed by a vascular neurologist. Stroke etiology was sub-classified as **Atherosclerotic** if the stroke etiology was thought to be due to intra- or extra-cranial atherosclerotic disease (ICAD and/or ECAD, respectively). **Non-atherosclerotic stroke** cases lacked atherosclerotic components.

**Figure 1.**
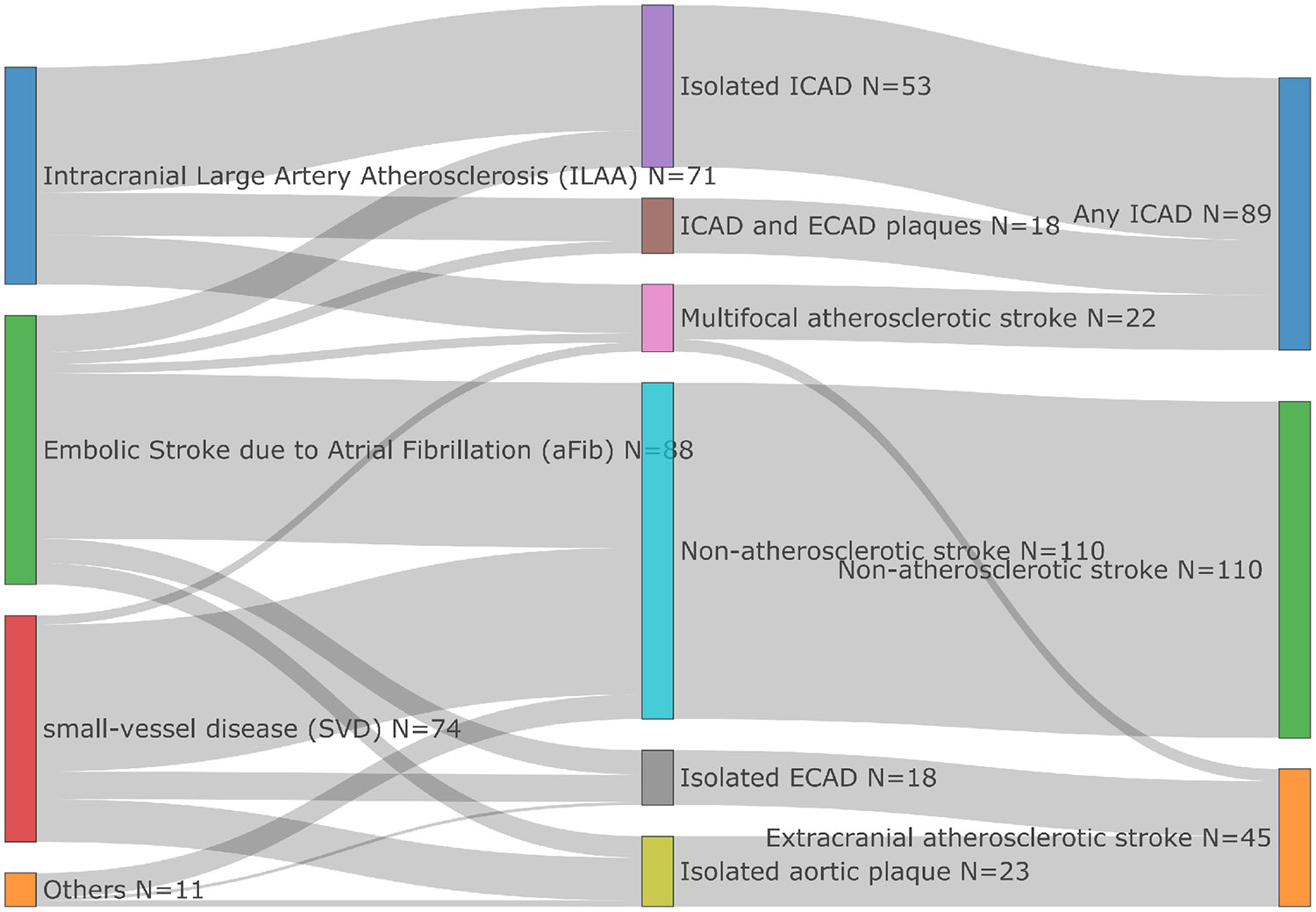
Acute ischemic stroke subtype classification in study population. ICAD: intracranial atherosclerotic disease; ECAD: extracranial atherosclerotic stroke.

Within the **Atherosclerotic** group of cases, **any ICAD** refers to the group in which ischemic incidents are due to ICAD or jointly caused by ICAD; readers should be aware that this is a broader definition than the standard ICAD term. **ECAD** refers to a subgroup that lacks ICAD elements. Our 244 AIS cases were divided into: any ICAD (*N* = 89)-Blue, ECAD (*N* = 45)-Orange and non-atherosclerotic (*N* = 110)-Green, Figure 1 (right side).

### 2.3 Lipid and apolipoprotein B100 measurement

Lipid levels [total Cholesterol (C), HDL-C, triglycerides] were measured via standardized enzymatic methods on an Integra400 plus Analyzer (Roche Diagnostics Corporation) by the Biomarkers Laboratory of the Irving Clinical and Translational Research Center, Columbia University Medical Center, NY. Plasma LDL-C levels were estimated with the NIH equation 2^[37]^. Plasma APOB levels were measured by a human enzyme-linked immunosorbent assay (ELISA), purchased from Mabtech (3715-HP-2), Inc. Cincinnati, OH. The percentage of APOB in Lp(a) particles was calculated by converting our APOB100 plasma levels from mg/dl to nmol/l and assuming a one-to-one ratio with Lp(a) levels in nmol/l, calculating the percentage. This serves as a rough estimate of how many particles are being carried by the Lp(a) particles in plasma; other studies have performed more thorough estimations of these calculations^[38]^ that includes corrections for plasma triglycerides, LDL-C, and non-HDL-C, which we did not do in our small cohort.

### 2.4 Lp(a) concentration and APO(a) isoform size

Lp(a) plasma concentrations were measured using the isoform-independent sandwich ELISA developed by the Northwest Lipid Metabolism and Diabetes Research Laboratory. In addition, the lab utilized gel electrophoresis to measure APO(a) isoform size. 250 μL of plasma was diluted in 40 μl of saline and combined with reducing buffer. The sample was then loaded onto an agarose gel, run overnight at 123V and 4 °C, transferred to a nitrocellulose membrane, immunoblotted, and imaged using the ChemiDoc MP Imaging System (Figure S1). This determined the isoform size present in the samples by comparison to an in-house standard (combined material containing six APO(a) isoforms, 38, 32, 24, 19, 15, and 12 KIV-2 repeats). The expression of each isoform was established using the Image Lab software, which calculated relative proportions of the two isoforms based on the intensity profile of each lane. This method has an intra-sample variability that does not exceed 15%^[39]^. A weighted isoform size (*wIS*) measurement was calculated^[40]^.

### 2.5 Measurement of OxPL

OxPL-APOB was measured with an immunoassay that uses the immunoglobulin M antibody E06, which binds to phosphocholine-containing phospholipids that share a common feature of a phosphocholine headgroup in a non-native geometric configuration. However, it remains unclear whether the OxPL-APOB assay captures the myriad of other oxidized phospholipids that trigger inflammatory signaling pathways in the various cell types involved in atherosclerosis^[41]^. Additionally, measurements of OxPL on Lp(a), using an APO(a) antibody and E06, were also performed at the University of California San Diego. The levels of OxPL-APO(a) represent oxidized phospholipids on Lp(a) particles, not limited to APO(a).

### 2.6 Data Analysis

Clinical characteristics are reported as absolute and percentage. Variables that were normally distributed are reported in mean and standard deviation format, while those that were not normally distributed are reported as medians along with interquartile ranges. The Wilcoxon Rank-Sum test (Mann-Whitney *U* test) was used to compare groups, as most of the biomarker values were right-skewed. For multi-group comparisons, an additional Benjamini-Hochberg false discovery rate correction was deployed on pairwise Mann-Whitney U test results. The acute stroke categories have a multi-level nature, therefore two Mann-Whitney U tests were rendered for the following: 1) in all the acute ischemic population, the biomarkers distribution in the atherosclerotic stroke group was contrasted with the non-atherosclerotic stroke group; 2) the biomarkers’ distribution in any ICAD group was compared to the biomarkers in extracranial atherosclerosis stroke group.

Based on the underlying hypothesis that abnormal lipid metabolism relates to atherosclerosis, a multivariate logistic regression model with 1) atherosclerotic stroke in atherosclerotic group versus non-atherosclerotic group, and 2) any ICAD versus ECAD group as the dependent variable was built with lipid biomarkers as the main exposure and age, sex, race/ethnicity, vascular risk factors (namely systolic blood pressure, blood glucose, HDL, LDL-C, and triglycerides), statin therapy, smoking history, and diabetes mellitus history were used as covariates. These variables were potential confounders based on plausibility and published studies showing effects on Lp(a) concentrations. In the regression model, all variables not following a normal distribution are logged with base 2. A *p*-value = 0.05 was used as the threshold establishing statistical significance. Data analysis was performed using R 4.4.1. The power analysis for the coefficient of interest in logistic regression was performed using R package pwrss 0.3.1^[42]^. The corresponding authors had full access to all the data and took responsibility for its integrity and the data analysis.

## 3. Results

The demographic and clinical characteristics of the study population (*N* = 244) are presented in Table 1. The median age of the participants was 73 years old. The population consisted of Non-Hispanic White, Non-Hispanic Black, Hispanic, and other (multiple or unknown backgrounds). Self-reported medical history was also incorporated into our data and highlights a population with an increased risk for multiple co-morbidities that drive ASCVD.

### 3.1 Distribution of biomarkers in study population

**Figure 2.**
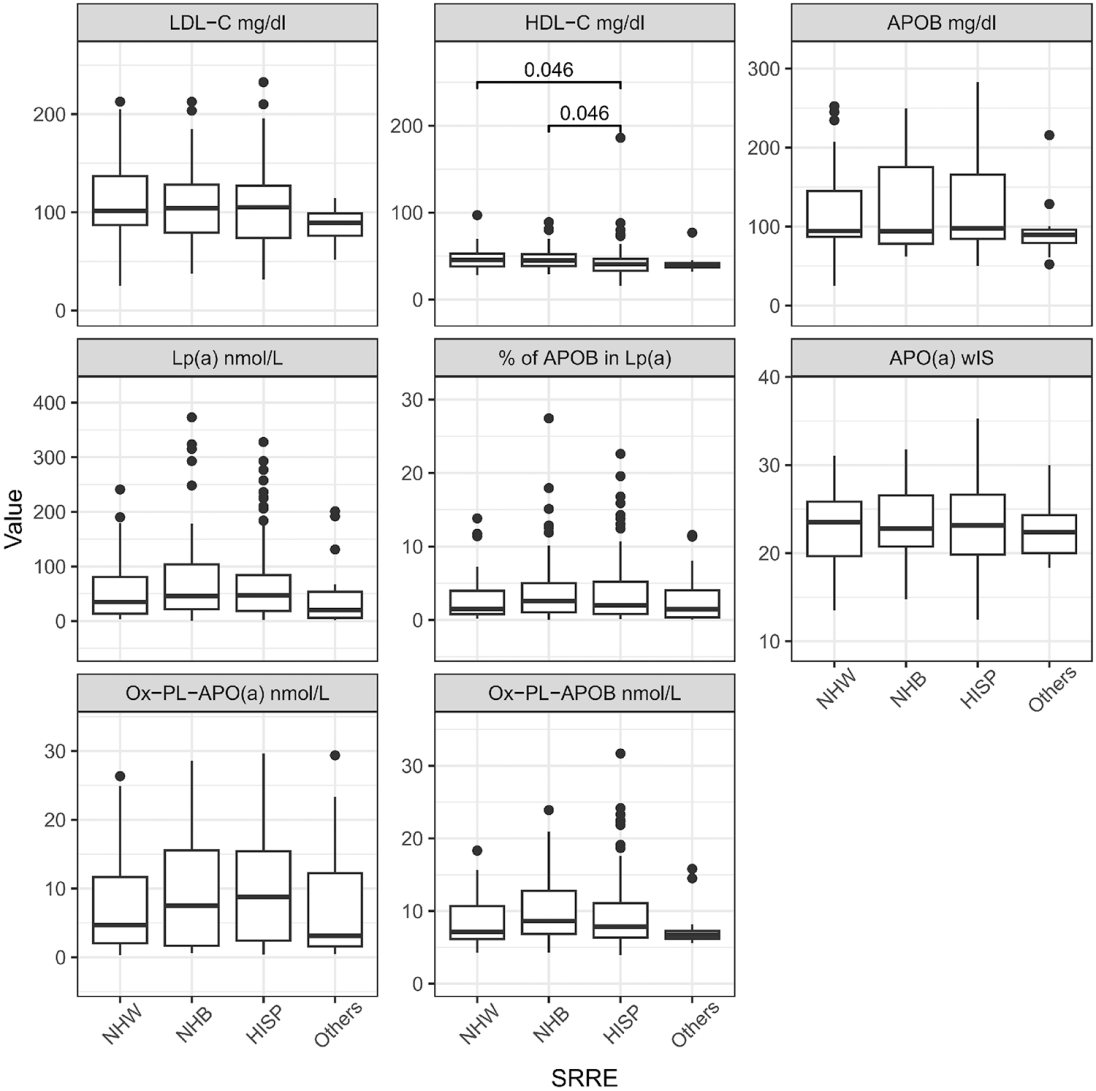
Lipid, lipoproteins and oxidized phospholipids levels by race/ethnicity. Significance in distribution comparison is assessed by pairwise Mann-Whitney U test with Benjamini-Hochberg FDR correction. Only significant results are illustrated. The source data can be found in Table S1. NHW: Non-Hispanic White; NHB: Non-Hispanic Black; HISP: Hispanic population; Chol: cholesterol; TG: triglyceride; HDL: high density lipoprotein; LDL: low density lipoprotein; APOB: apolipoprotein B100; APO(a): apolipoprotein (a); Lp(a): lipoprotein(a); OxPL: oxidized phospholipids; *wIS*: weighted isoform size; other: multiple backgrounds or unknown; FDR: false discovery rate.

The distribution of lipid-related biomarkers varied systematically across the ethnic groups (Table 2, Figure 2, Table S1). Across all participants, the median Lp(a) level was 41.9 nmol/L, with significant variability observed across different demographic groups. As expected, Non-Hispanic Blacks and Hispanics exhibited the highest median Lp(a) levels at 45.9 nmol/L (21.56-103.71) and 46.87 nmol/L (18.65-84.36), respectively. LDL-C levels were also comparable between Non-Hispanic Whites (110.09 mg/dL) and Blacks (108.76 mg/dL), and slightly higher than in Hispanics (105.99 mg/dL). Hispanics had the highest median TG levels at 117 mg/dL (88-169), which was higher compared to other groups.

The percentage of APOB in Lp(a) was highest in Non-Hispanic Blacks at 2.57% (1.05-5), which was notably higher compared to Non-Hispanic Whites at 1.51% (0.78-3.96). Hispanic and Non-Hispanic Black individuals had the highest median OxPL-APO(a) levels [8.76 nmol/L (2.41-15.41); 7.52 nmol/l (1.65-15.54), respectively], which has been linked to higher oxidative stress, yet no differences were observed in OxPL-APOB. In this AIS cohort, the APO(a) isoform sizes, expressed as w*IS,* were small (mean of 23) and no differences were noted between the different ethnic groups. HDL-C levels in the Hispanic population were significantly lower than the Non-Hispanic Blacks and White groups.

### 3.2 Associations of biomarkers in AIS atherosclerotic *vs.* non-atherosclerotic groups

**Figure 3.**
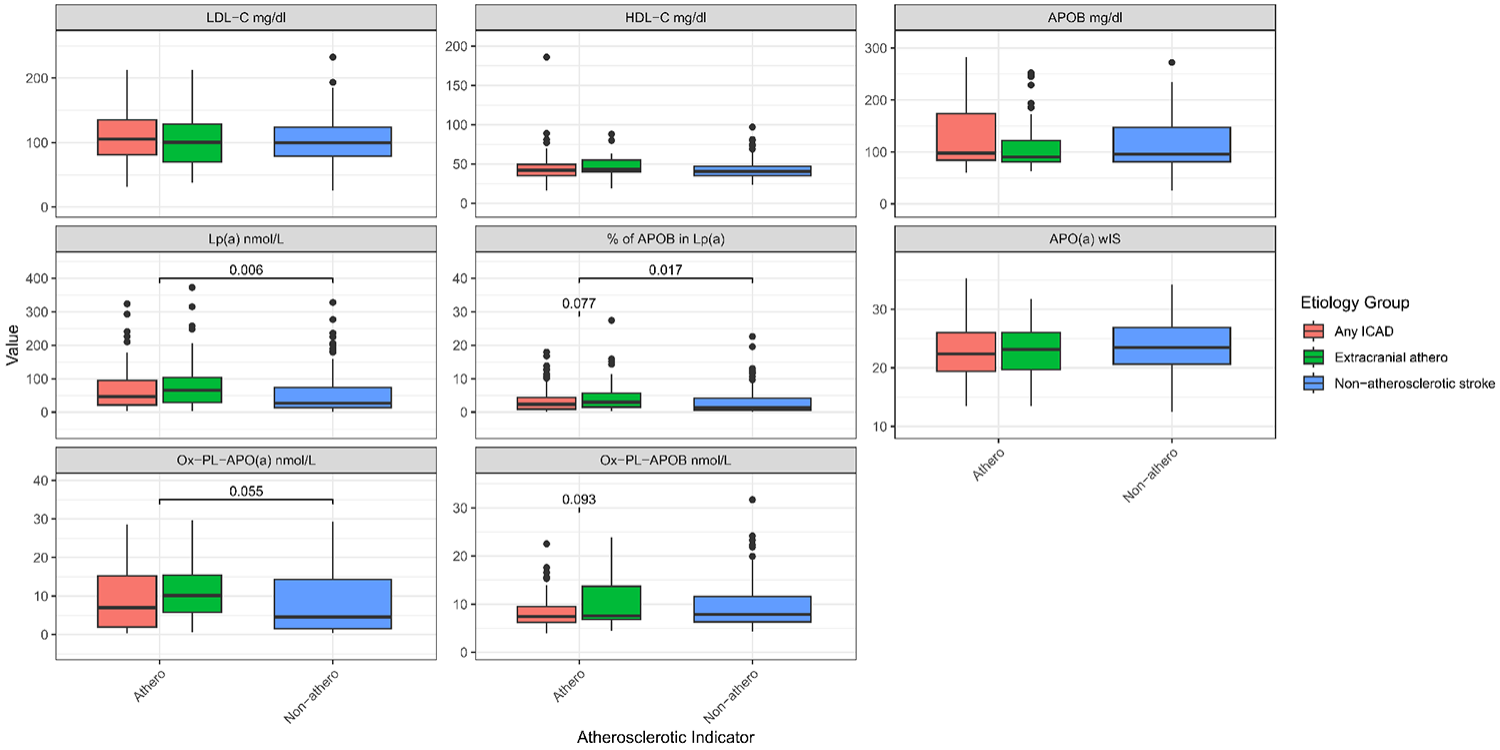
Boxplot for lipid, lipoproteins and oxidized phospholipids levels by stroke etiology classifications. Chol: Cholesterol; TG: triglyceride; HDL: high density lipoprotein; LDL: low density lipoprotein; APOB: apolipoprotein B100; APO(a): apolipoprotein (a); Lp(a): lipoprotein(a); OxPL: oxidized phospholipids; *wIS*: weighted isoform size; other: multiple backgrounds or unknown; Significance in distribution comparison is assessed by Mann-Whitney U test; Only significant (*p* < 0.05) and trend (*p* < 0.10) results are illustrated; The source data can be found in Table S2.

The associations of Lp(a) and other biomarkers were examined in the AIS atherosclerosis *vs.* non-atherosclerosis groups (Figure 3, Table 3, Table S2). Participants with ECAD showed the highest median Lp(a) levels at 66.05 nmol/L (29.57-103.79). Those without atherosclerosis had significantly (*p* = 0.006) lower median Lp(a) levels, 27.12 nmol/L (13.38-73.95).

The highest total cholesterol and LDL-C levels were observed in participants with ICAD, with total cholesterol at 177.06 mg/dL and LDL-C at 111.15 mg/dL. HDL-C levels were consistent across groups, with a slight increase in those with ECAD (median 43 mg/dL). Similarly, TG levels were uniform across all groups, with slight variations, indicating that TG might have less impact on the type of atherosclerosis present in this cohort.

The percentage of APOB present in Lp(a) was highest in the ECAD group at 2.98% (1.47-5.72), which showed a higher trend (*p*-value = 0.077) when compared with the ICAD group, while the atherosclerotic group including both the ICAD and ECAD was significantly higher (*p*-value = 0.017) when compared with the non-atherosclerotic group, potentially indicating a relationship between APOB content in Lp(a) and extracranial atherosclerosis. Additionally, in the ECAD group there was a higher median OxPL-APO(a) levels (10.15 nmol/L), suggesting higher oxidative stress in this group.

### 3.3 Association of biomarkers in AIS atherosclerotic groups

To further investigate the relationship of these biomarkers across the different stroke etiology categories, we performed logistic regression. We examined the associations between biomarkers in the different atherosclerotic groups [*N* = 114 [any ICAD (89) and ECAD (45)] *vs. N* = 110 non-atherosclerotic stroke (Figure 1)] (Table 4, Table S3). Lp(a) levels, when 2-based logged, were significantly associated with an increased risk of stroke, with an OR of 1.30 and a statistically significant *p*-value of 0.0057, highlighting the role of Lp(a) as a strong risk factor in stroke caused by atherosclerotic factors. APO(a) *wIS* shows an OR of 0.94 with a *p*-value of 0.079, implying a potential but not statistically significant protective effect against stroke.

There was no statistical association of APOB with stroke risk (OR = 0.91, *p*-value = 0.77). However, the percentage of APOB in Lp(a), when 2-based logged, showed a significant association with stroke risk (OR = 1.29, *p*-value = 0.0069). OxPL-APO(a), when 2-based logged, is associated with an increased stroke risk from atherosclerotic factors (OR = 1.27, *p*-value = 0.017). However, OxPL-APOB (OR = 0.91, *p*-value = 0.76) was not significantly associated with stroke risk.

Additional analysis of the atherosclerotic stroke sub-groups [ICAD (*N* = 89) *vs.* ECAD (*N* = 45)] showed that the percentage of APOB in Lp(a) (OR = 0.62, *p*-value = 0.007), Lp(a) (OR = 0.66, *p*-value = 0.019), and OxPL-APO(a) (OR = 0.69, *p*-value = 0.04) were associated with a lower odds to ICAD stroke within atherosclerotic cohorts (Table 5, Table S4).

### 3.4 Association of biomarkers in AIS atherosclerotic subgroups with non-atheroclerotic group

Considering the systematic differences in biomarkers that were observed between ICAD and ECAD atherosclerotic groups, we compared them separately to the non-atherosclerotic group. We observed that a two-fold increase in Lp(a) concentration is associated with higher odds of ECAD when compared to the non-atherosclerotic group (OR = 1.53, *p*-value = 0.0027), and observed two-fold increases in the percentage of APOB in Lp(a) (OR = 1.54, *p*-value = 0.002) and OxPL-APO(a) (OR = 1.53, *p*-value = 0.0076) in the ECAD group when compared to the non-atherosclerotic group (Table 6, Table S5). The finding on Lp(a) level aligned with the finding on the smaller APO(a) isoform size (OR = 0.89, *p* = 0.045). However, if we compared the ICAD only group *vs.* non-atherosclerotic stroke populations, no associations were found (Table S6). The stratified analysis suggests ECAD as having a larger association with biomarkers in those with high Lp(a).

Due to our small sample size, power analysis for all the study outcome data presented on Table 4, Table 5, Table 6, and non-significant findings, are presented on Table S3,S4,S5,S6, respectively. These limited powers support the need for larger studies that can serve as validation or counter statements to our negative study findings like on APOB concentration and scenarios, like Table S6. The provided 80% power sample size estimation can assist in future study design.

## 4. Discussion

In this cross-sectional analysis of AIS subgroups, we observed expected lipid differences across ethnicities, including higher median Lp(a) levels among Non-Hispanic Black and Hispanic participants. Consistent with prior reports linking small APO(a) isoform sizes to stroke risk^[7,43]^, our AIS cohort demostrated small APO(a) sizes. High Lp(a) levels have also been implicated in stroke among younger adults^[16]^; however our current cohort was composed of elderly adults. Studies in a younger cohort could provide insightful data on whether these biomarkers could be useful in that population.

While previous studies have established elevated Lp(a) as a significant risk factor for ischemic stroke, particularly in specific populations^[7,15,44,45]^, we aimed to further explore associated biomarkers that may refine atherosclerotic stroke classification. In alignment with prior meta-analysis^[46]^, we found that Lp(a) levels were positively associated with atherosclerotic stroke. Specifically, higher proportions of APOB on Lp(a), total serum Lp(a), and OxPL–APO(a) concentrations showed a greater association with ECAD than with ICAD. These results reinforce a potential role for Lp(a) and its oxidative modifications in the pathogenesis of large-artery stroke^[46,47]^. *In vitro* studies using human samples have shown that Lp(a) can be the main carrier of OxPL. Extracranial vascular beds, particularly the coronary, carotid, and aortic arteries, are large elastic arteries with structural and biological features that make them vulnerable to OxPL-mediated vascular injury^[9,32,48,49]^. Notably, a smaller study in AIS found higher Lp(a) levels among patients with unstable compared to stable plaque^[50]^, suggesting that this subgroup may be particularly relevant for evaluating the biomarkers assessed here.

Our findings support previous work identifying APO(a) as the primary carrier of OxPL and a key driver of atherosclerosis^[48,49]^. The observed correlations between Lp(a)-OxPL and smaller apo(a) isoforms further suggest that oxidative modifications of Lp(a) may contribute to vascular instability and stroke risk^[51]^. In contrast, APOB and OxPL–APOB were not significantly associated with atherosclerotic stroke in our study, reflecting the high statin use (45%) within this cohort, although analyses were adjusted for this variable. Importantly, other studies in larger cohorts have reported associations between APOB and OXPL-APOB with stroke risk^[31,48,52,53]^.

Elevated total cholesterol and LDL-C concentrations in the ICAD subgroup underscore the importance of intensive lipid management to prevent intracranial atherosclerosis. The specific association between high Lp(a) and ECAD observed here suggests that additional work is required in this area to see if targeted Lp(a)-lowering therapies may hold promise for this subgroup. The MESA studies diverse cohort is comparable to the current study participants. While prior MESA data linked elevated Lp(a) to coronary heart disease but not to ischemic stroke^[54]^, our findings highlight the potential contribution of OxPL-related mechanisms, which were not assessed in that study. Consistent with subsequent MESA analyses^[55]^, we observed higher OxPL levels in the atherosclerotic subgroup. In summary, the proportion of APOB carried on Lp(a) and concentrations of OxPL–apo(a) appear to provide additional mechanistic insight into the etiology and risk stratification of AIS. These biomarkers may help refine stroke classification and identify therapeutic targets within the atherosclerotic spectrum of disease.

## 5. Study Limitations

Despite detailed characterization of the AIS subgroups, our study lacked a control group, which would have strengthened comparative analyses. Our sample size was small, with statistical power being low for some of the outcomes, the latter limiting the ability to perform stratified analyses by tertiles or quartiles. We have included a power analysis, along with an expected 80% power sample size for each outcome in the supplemental data based on Monte-Carlo resampling. Replication of the findings with these biomarkers in independent stroke cohorts will be useful for a more robust conclusion about our negative findings. Additionally, plasma samples were stored for less than five years before Lp(a) measurement, but storage duration may have modestly affected measured concentrations and *wIS*. Prior reports suggest that Lp(a) degradation during long-term storage occurs in a heterogeneous and non-linear manner. All samples in our study were frozen and never thawed, reducing but not eliminating this potential bias. Finally, although samples were obtained between 48 hours and seven days after the acute event, variability in sampling time may have influenced measured biomarker levels.

## Supplementary Material

Supplementary materials

The supplementary material for this article is available at: Supplementary materials.

## Figures and Tables

**Table 1.** Study population: Demographic and clinical characteristics.

Variables		*N* = 244
**Age**		73(61-82)
**Sex**	Male	141(58%)
	Female	103(42%)
**Ethnicity**	Non-Hispanic white individuals	51(21%)
	Non-Hispanic Black individuals	52(21%)
	Hispanic individuals	125(51%)
	Other-multiple background or unknown	16(7%)
**Hypertension**		196(80%)
**Antihypertensive medication use**		178(73%)
**Diabetes Mellitus**		101(41%)
**Dyslipidemia**		141(58%)
**Statin therapy**		111(45%)
**Smoking history**		44(18%)
**Coronary artery disease**		66(27%)

Data reported as median [IQR] for continuous variables and number (%) for categorical variables; *N*: number of participants; IQR: interquartile range.

**Table 2.** Lipid, lipoproteins, oxidized phospholipids levels.

Biomarkers	All Participants (*N* = 244)	Non-Hispanic White (*N* = 51)	Non-Hispanic Black (*N* = 52)	Hispanic (*N* = 125)	Other (*N* = 16)
Total-Chol mg/dl	172.22 ± 46.68	176.67 ± 45.57	176.4 ± 48.55	171.19 ± 48.04	151.77 ± 24.88
TG mg/dl	106 (80.75-156.25)	93 (75.75-139.25)	88 (65.25-133.75)	117 (88-169)	103 (77-157)
HDL-C mg/dl	42 (35-49)	45.5 (38-52.75)	45 (38.5-52)	40.5 (33.25-46.75)	40 (37-42)
LDL-C mg/dl	105.99 ± 39.93	110.09 ± 41.01	108.76 ± 41.79	105.47 ± 40.39	87.41 ± 18
Lp(a) nmol/L	41.9 (18.08-92.17)	34.97 (13.73-80.74)	45.9 (21.56-103.71)	46.87 (18.65-84.36)	20.25 (5.7-53.5)
APOB mg/dl	95.68 (81.82-155.33)	94.4 (86.91-144.86)	93.97 (78.38-175.31)	97.6 (84.49-165.75)	89.56 (79.15-96.1)
% of APOB in Lp(a)	1.94 (0.81-4.86)	1.51 (0.78-3.96)	2.57 (1.05-5)	1.98 (0.81-5.19)	1.45 (0.37-4.04)
APO(a) *wIS*	23.28 ± 4.36	22.98 ± 4.02	23.39 ± 4.18	23.43 ± 4.69	22.79 ± 3.41
OxPL-APO(a) nmol/L	6.86 (1.95-15.27)	4.68 (2.03-11.66)	7.52 (1.65-15.54)	8.76 (2.41-15.41)	3.1 (1.58-12.24)
OxPL-APOB nmol/L	7.71 (6.35-11)	7.13 (6.14-10.67)	8.62 (6.86-12.79)	7.84 (6.36-11.08)	6.7 (6.22-7.25)
Smaller APO(a) isoform Size	19.67 ± 4.04	19.69 ± 3.75	19.71 ± 4.01	19.6 ± 4.18	20.06 ± 4.3
Larger APO(a) isoformSize	27.09 ± 5.43	27.02 ± 4.72	27.21 ± 5.29	26.93 ± 5.87	28.2 ± 4.68

All data presented as medians and IQR. IQR: interquartile range; Chol: cholesterol; TG: triglyceride; HDL: high density lipoprotein; LDL: low density lipoprotein; APOB: apolipoprotein B100; APO(a): apolipoprotein (a); Lp(a): lipoprotein(a); OxPL: oxidized phospholipids; *wIS*: weighted isoform size; Isoform Size: means of the smaller and larger isoform size obtained via gel electrophoresis; other: multiple backgrounds or unknown; Normally distributed features are in means(s.d.) format, while the skewed distribution is shown in median (IQR) format.

**Table 3.** Lipid, lipoproteins and oxidized phospholipids levels by new etiology classifications.

Biomarkers	All Participants	Any ICAD	Extracranial atherosclerotic	Non-atherosclerotic
Total-Chol mg/dl	172.22 ± 46.68	177.06 ± 48.05	172.56 ± 46.52	168.12 ± 45.73
TG mg/dl	106 (80.75-156.25)	98 (80.5-163)	108 (80-136)	108 (81.75-157)
HDL-C mg/dl	42 (35-49)	42 (35-49.5)	43 (40-55)	40.5 (35-47)
LDL-C mg/dl	105.99 ± 39.93	111.15 ± 41.57	105.37 ± 41.42	102.05 ± 37.82
Lp(a) nmol/L	41.9 (18.08-92.17)	46.64 (21.65-94.54)	66.05 (29.57-103.79)	27.12 (13.38-73.95)
APOB mg/dl	95.68 (81.82-155.33)	97.72 (84.16-173.59)	90.34 (81.14-121.82)	95.6 (81.23-147.36)
% of APOB in Lp(a)	1.94 (0.81-4.86)	2.38 (0.88-4.34)	2.98 (1.47-5.72)	1.38 (0.67-4.16)
APO(a) *wIS*	23.28 ± 4.36	22.79 ± 4.24	23.19 ± 4.07	23.72 ± 4.55
OxPL-APO(a) nmol	6.86 (1.95-15.27)	6.96 (1.98-15.28)	10.15 (5.75-15.47)	4.53 (1.48-14.33)
OxPL-APOB nmol	7.71 (6.35-11)	7.48 (6.22-9.49)	7.6 (6.84-13.71)	7.86 (6.34-11.63)
Smaller APO(a) isoform size	19.67 ± 4.04	19.6 ± 4.43	18.8 ± 3.54	20.09 ± 3.88
Larger APO(a) isoform size	27.09 ± 5.43	26.65 ± 5.34	27 ± 5.48	27.49 ± 5.51

Normally distributed features are in means ± s.d. format, while the skewed distribution is shown in median (IQR) format. IQR: interquartile range; Chol: cholesterol; TG: triglyceride; HDL: high density lipoprotein; LDL: low density lipoprotein; APOB: apolipoprotein B100; APO(a): apolipoprotein (a); Lp(a): lipoprotein(a); OxPL: oxidized phospholipids; *wIS*: weighted isoform size; Isoform Size: means of the smaller and larger isoform size obtained via gel electrophoresis; other: multiple backgrounds or unknown.

**Table 4.** Associations of biomarkers with the presence of atherosclerotic stroke.

Biomarkers	Effect Size	Odds Ratio	*p*-value
Lp(a)(nmol/l), 2-based logged	0.26	1.30^†^	0.0057*
APOB (mg/dl), 2-based logged	−0.09	0.91^†^	0.77
% of APOB in Lp(a)	0.08	1.08	0.087’
% of APOB in Lp(a), 2-based logged	0.25	1.29^†^	0.0069*
APO(a) *wIS*	−0.06	0.94	0.079’
Smaller APO(a) isoform	−0.05	0.95	0.17
OxPL-APO(a) (nmol/L), 2-based logged	0.24	1.27^†^	0.017*
OxPL-APOB (nmol/L), 2-based logged	−0.10	0.91^†^	0.76

* statistical significance

’ statistical trending; the statistical significance threshold is 0.05 in logistic regression

† when the biomarker is 2-based logged, the odds ratio is associated with the doubling of biomarker rather than 1-unit increase; Lp(a): Lipoprotein(a); OxPL: oxidized phospholipid; APOB: apolipoprotein B100; APO(a): apolipoprotein (a); *wIS*: weighted isoform size; The power analysis is in Table S3.

**Table 5.** Associations of biomarkers with the presence of any ICAD-related atherosclerotic stroke.

Biomarkers	Effect Size	Odds Ratio	*p*-value
Lp(a)(nmol/l), 2-based logged	−0.41	0.66^†^	0.019*
APOB (mg/dl), 2-based logged	0.84	2.31^†^	0.10
% of APOB in Lp(a)	−0.14	0.87	0.011*
% of APOB in Lp(a), 2-based logged	−0.47	0.62^†^	0.007*
APO(a) *wIS*	−0.02	0.98	0.66
Smaller APO(a) isoform	0.08	1.08	0.15
OxPL-APO(a) (nmol/L), 2-based logged	−0.38	0.69^†^	0.04*
OxPL-APOB (nmol/L), 2-based logged	−0.95	0.39^†^	0.054’

* statistical significance

’ statistical trending; the statistical significance threshold is 0.05 in logistic regression

† when the biomarker is 2-based logged, the odds ratio is associated with the doubling of biomarker rather than 1-unit increase; Lp(a): Lipoprotein(a); OxPL: oxidized phospholipid; APOB: apolipoprotein B100; APO(a): apolipoprotein (a); *wIS*: weighted isoform size; The power analysis is in Table S4.

**Table 6.** Associations of biomarkers with the presence of ECAD *vs.* non-atherosclerotic stroke population.

Biomarkers	Effect Size	Odds Ratio	*p*-value
Lp(a)(nmol/l), 2-based logged	0.43	1.53^†^	0.0027*
APOB (mg/dl), 2-based logged	−0.6	0.55^†^	0.19
% of APOB in Lp(a)	0.12	1.13	0.019*
% of APOB in Lp(a), 2-based logged	0.43	1.54^†^	0.002*
APO(a) *wIS*	−0.06	0.94	0.23
Smaller APO(a) isoform	−0.12	0.89	0.045*
OxPL-APO(a) (nmol/L), 2-based logged	0.43	1.53^†^	0.0076*
OxPL-APOB (nmol/L), 2-based logged	0.26	1.30^†^	0.51

* statistical significance (the statistical significance threshold is 0.05 in logistic regression)

† when the biomarker is 2-based logged, the odds ratio is associated with the doubling of biomarker rather than 1-unit increase; Lp(a): Lipoprotein(a); OxPL: oxidized phospholipid; APOB: apolipoprotein B100; APO(a): apolipoprotein (a); *wIS*: weighted isoform size; ECAD: extracranial atherosclerotic stroke ; The power analysis is in Table S5.
