# Supplementary material for: Association of lipoprotein(a), oxidized phospholipids and apolipoprotein B100 in acute ischemic stroke cohort
Source: Adv Lipoprotein A Res. Author manuscript; Available in PMC 2026 Feb 26. (PMC12933386; doi:10.70401/alr.2026.0005)
Supplement: Supplementary materials [file NIHMS2140882-supplement-Supplementary_materials.pdf]

Advances in Lipoprotein(a) Research
https://doi.org/10.70401/alr.2025.0005

Supplementary information

Association of lipoprotein(a), oxidized phospholipids and apolipoprotein B100 in acute ischemic stroke cohort

Yihao Li, Emmie Then, Salwa Rahman, Nelsa Matienzo, Anastasiya Matveyenko, Marianna Pavlyha, Farid Khasiyev, Randolph S Marshall, Joshua Willey, Jose Gutierrez, Gissette Reyes-Soffer

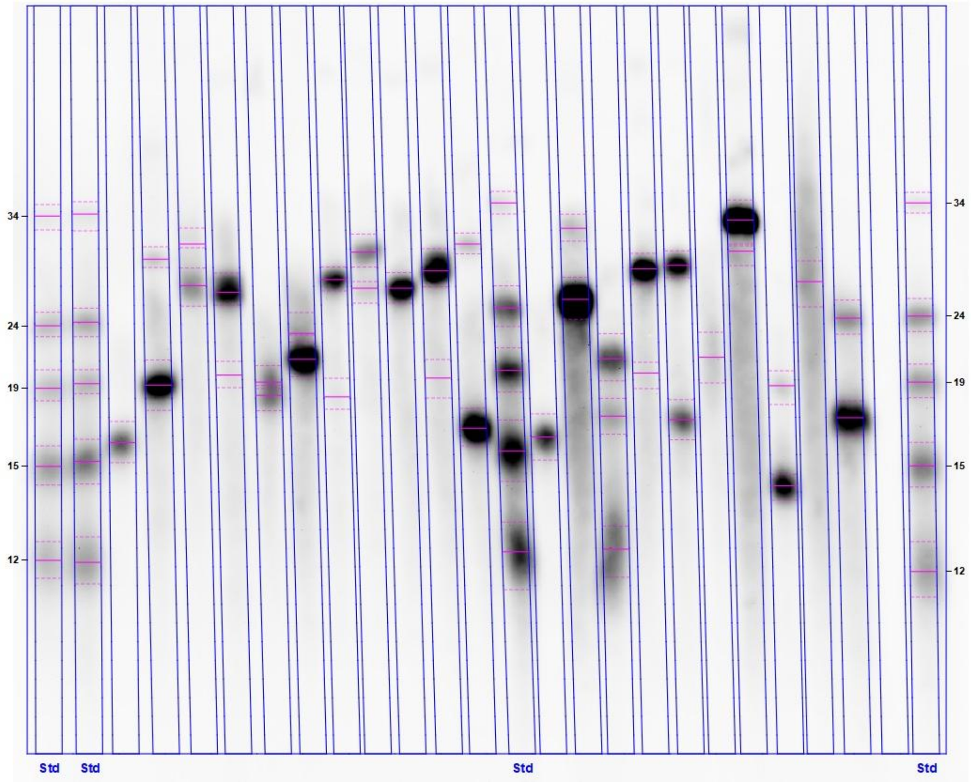

Figure S1. Immunoblots of APO(a) isoforms. Representative immunoblot showing lipoprotein(a) [Lp(a)] APO(a) isoforms separated on agarose gel electrophoresis. Plasma samples from individual subjects were electrophoresed in parallel lanes alongside standards, which are shown on both sides of the gel. APO(a) isoforms are visualized as discrete bands migrating between ~12 and 34 kDa based on the number of kringle IV type-2 (KIV-2) repeats.

Table S1. Pairwise Mann-Whitney U test result for biomarkers across races.

**Biomarkers**	NHB *vs.* HISP	NHW *vs.* HISP	HISP *vs.* Other	NHB *vs.* HISP	NHB *vs.* Other	NHW *vs.* Other
Total-Chol mg/dl	0.719	0.719	0.196	0.983	0.196	0.196
TG mg/dl	0.017*	0.109	0.428	0.428	0.428	0.751
HDL-C mg/dl	0.046*	0.046*	0.928	0.928	0.168	0.168
LDL-C mg/dl	0.920	0.920	0.151	0.920	0.151	0.151
Lp(a) nmol/L	0.483	0.453	0.190	0.290	0.190	0.290
APOB mg/dl	0.783	0.783	0.252	0.783	0.312	0.252
% of APOB in Lp(a)	0.490	0.490	0.490	0.490	0.490	0.490
APO(a) wIS	0.887	0.887	0.887	0.887	0.887	0.887
Smaller APO(a) isoform size	0.946	0.935	0.935	0.935	0.935	0.935
Ox-PL-APO(a) nmol/L	0.830	0.830	0.830	0.830	0.830	0.830
Ox-PL-APOB nmol/L	0.281	0.281	0.281	0.137	0.137	0.669

NHW: Non-Hispanic White; NHB: Non-Hispanic Black; HISP: Hispanic population; Other: multiple backgrounds or unknown; Chol: cholesterol; TG: triglyceride; HDL:high density lipoprotein; LDL: low density lipoprotein; APO: apolipoprotein; Lp(a): lipoprotein(a); OxPL: oxidized phospholipids; *wIS*: weighted isoform size; *: statistically significant; Significance in distribution comparison is tested by pairwise Mann-Whitney U test with Benjamini-Hochberg FDR correction.

Table S2. Mann-Whitney U test result for biomarkers across etiology groups.

**Biomarkers**	Any ICAD *vs.* ECAD	Athero *vs.* Non-athero
Total-Chol mg/dl	0.743	0.220
TG mg/dl	0.877	0.604
HDL-C mg/dl	0.179	0.225
LDL-C mg/dl	0.463	0.269
Lp(a) nmol/L	0.125	0.006*
APOB mg/dl	0.172	0.560
% of APOB in Lp(a)	0.077'	0.017*
APO(a) wIS	0.517	0.148
Smaller APO(a) isoform size	0.474	0.061'
Ox-PL-APO(a) nmol/L	0.157	0.055'
Ox-PL-APOB nmol/L	0.093'	0.683

Any ICAD: atherosclerotic stroke solely or jointly caused by intracranial atherosclerotic stroke; ECAD: extracranial atherosclerotic stroke; Athero: atherosclerotic stroke; Non-athero: nonatherosclerotic stroke; Chol: cholesterol; TG: triglyceride; HDL: high density lipoprotein; LDL: low density lipoprotein; APO: apolipoprotein; Lp(a): lipoprotein(a); OxPL: oxidized phospholipids; *wIS*: weighted isoform size; *: statistical significant ($p < 0.05$); ': statistical trend ($p < 0.10$); Significance in distribution comparison is tested by Mann-Whitney U test.

Table S3. Associations of biomarkers with the presence of atherosclerotic stroke with power analysis.

**Biomarkers**	Effect Size	OR	*P*-value	current power	N_obs	80% power size
Lp(a) nmol/L, 2-logged	0.26	1.30†	0.0057*	63.11%	203	299
APOB mg/dl, 2-logged	-0.09	0.91†	0.77	6.19%	197	14,688
% of APOB in Lp(a)	0.08	1.08	0.087’	52.76%	197	369
% of APOB in Lp(a), 2-logged	0.25	1.29†	0.0069*	81.19%	197	192
APO(a) *wIS*	-0.06	0.94	0.079’	34.73%	203	636
Smaller APO(a) isoform	-0.05	0.95	0.17	26.37%	203	888
Ox-PL-APO(a) nmol, 2-logged	0.24	1.27†	0.017*	66.46%	176	240
Ox-PL-APOB nmol, 2-logged	-0.10	0.91†	0.76	6.52%	176	10,284

*: statistical significance, ’: statistical trending (the statistical significance threshold is 0.05 in logistic regression); †: when the biomarker is 2-based logged, the odd ratio is associated with the doubling of biomarker rather than 1-unit increase; Lp(a): lipoprotein(a); OxPL: oxidized phospholipid; APOB: apolipoprotein B100; APO(a): apolipoprotein (a); *wIS:* weighted isoform size. Current power: the power for the biomarkers corresponding coefficient; N_obs: the actual number of samples included in the model; 80% power size: the sample size required to obtain an 80% power, based on current distributions; The power analysis is implemented by R package pwrss.

Table S4. Associations of biomarkers with the presence of any ICAD-related atherosclerotic stroke in atherosclerotic cohort with power analysis.

**Biomarkers**	Effect Size	OR	*P*-value	current power	N_obs	80% power size
Lp(a) nmol/L, 2-logged	-0.41	0.66†	0.019*	69.33%	114	145
APOB mg/dl, 2-logged	0.84	2.31†	0.10	5.40%	111	24,019
% of APOB in Lp(a)	-0.14	0.87	0.011*	82.89%	111	104
% of APOB in Lp(a), 2-logged	-0.47	0.62†	0.007*	94.94%	111	72
APO(a) *wIS*	-0.02	0.98	0.66	8.12%	114	3,238
Smaller APO(a) isoform	0.08	1.08	0.15	18.32%	114	779
Ox-PL-APO(a) nmol, 2-logged	-0.38	0.69†	0.04*	76.23%	100	109
Ox-PL-APOB nmol, 2-logged	-0.95	0.39†	0.054’	33.50%	100	318

*: statistical significance, ’: statistical trending, the statistical significance threshold is 0.05 in logistic regression; †: when the biomarker is 2-based logged, the odd ratio is associated with the doubling of biomarker rather than 1-unit increase; Lp(a): lipoprotein(a); OxPL: oxidized phospholipid; APOB: apolipoprotein B100; APO(a): apolipoprotein (a); *wIS:* weighted isoform size; Current power: the power for the biomarkers corresponding coefficient; N_obs: the actual number of samples included in the model; 80% power size: the sample size required to obtain an 80% power, based on current distributions; The power analysis is implemented by R package pwrss.

Table S5. Associations of biomarkers with the presence of ECAD *vs.* non-atherosclerotic stroke population.

**Biomarkers**	Effect Size	OR	*P*-value	current power	N_obs	80% power size
Lp(a) nmol/L, 2-logged	0.43	1.53†	0.0027*	91.43%	129	95
APOB mg/dl, 2-logged	-0.6	0.55†	0.19	5.99%	124	10,875
% of APOB in Lp(a)	0.12	1.13	0.019*	75.52%	124	138
% of APOB in Lp(a), 2-logged	0.43	1.54†	0.002*	97.11%	124	71
APO(a) *wIS*	-0.06	0.94	0.23	13.49%	129	1,377
Smaller APO(a) isoform	-0.12	0.89	0.045*	17.17%	129	965
Ox-PL-APO(a) nmol, 2-logged	0.43	1.53†	0.0076*	93.40%	109	75
Ox-PL-APOB nmol, 2-logged	0.26	1.30†	0.51	12.83%	109	1,251

*: statistical significance, ': statistical trending, the statistical significance threshold is 0.05 in logistic regression; †: when the biomarker is 2-based logged, the odd ratio is associated with the doubling of biomarker rather than 1-unit increase; Lp(a): Lipoprotein(a); ECAD: extracranial atherosclerotic stroke; OxPL: oxidized phospholipid; APOB: apolipoprotein B100; APO(a): apolipoprotein (a); *wIS:* weighted isoform size; Current power: the power for the biomarkers corresponding coefficient; N_obs: the actual number of samples included in the model; 80% power size: the sample size required to obtain an 80% power, based on current distributions; The power analysis is implemented by R package pwrss.

Table S6. Associations of biomarkers with the presence of ICAD *vs.* non-atherosclerotic stroke population.

**Biomarkers**	Effect Size	OR	*P*-value	current power	N_obs	80% power size
Lp(a) nmol/L, 2-logged	0.15	1.16†	0.151	32.30%	163	552
APOB mg/dl, 2-logged	0.11	1.12†	0.754	6.42%	159	9,860
% of APOB in Lp(a)	0.03	1.04	0.467	12.82%	159	1,845
% of APOB in Lp(a), 2-logged	0.13	1.14†	0.192	29.48%	159	601
APO(a) *wIS*	-0.06	0.94	0.101	25.49%	163	739
Smaller APO(a) isoform	-0.02	0.98	0.61	8.26%	163	4,480
Ox-PL-APO(a) nmol, 2-logged	0.15	1.16†	0.169	32.66%	143	478
Ox-PL-APOB nmol, 2-logged	-0.38	0.68†	0.292	17.23%	143	1,070

None of the variables reached statistical significance threshold 0.05 in logistic regression; †: when the biomarker is 2-based logged, the odd ratio is associated with the doubling of biomarker rather than 1-unit increase; Lp(a): Lipoprotein(a); OxPL: oxidized phospholipid; APOB: apolipoprotein B100; APO(a): apolipoprotein (a); *wIS:* weighted isoform size; ICAD: intracranial atherosclerotic stroke; Current power: the power for the biomarkers corresponding coefficient; N_obs: the actual number of samples included in the model; 80% power size: the sample size required to obtain an 80% power, based on current distributions; The power analysis is implemented by R package pwrss.
